# Veganism, cuisine, and class: exploring taste as a facilitator in adopting a vegan lifestyle in Santiago, Chile

**DOI:** 10.3389/fsoc.2024.1356457

**Published:** 2024-04-16

**Authors:** Claudia Giacoman, Camila Joustra

**Affiliations:** ^1^Instituto de Sociología, Facultad de Ciencias Sociales, Pontificia Universidad Católica de Chile, Santiago, Chile; ^2^FONDECYT N°1201629, Santiago, Chile

**Keywords:** habitus, social class, veganism, taste, food, cultural dispositions, global south, Chile

## Abstract

**Introduction:**

Veganism is a movement that avoids consuming animal products. This lifestyle is commonly represented as elitist despite the broad range of people who follow it. Using Bourdieu's taste theory, this study analyzes how personal culinary tastes of different social classes generate favorable (or unfavorable) dispositions to adopting veganism.

**Methods:**

We analyzed 73 biographical interviews with 40 young vegans in three different waves.

**Results:**

The findings reveal that all social classes exhibit favorable dispositions towards veganism. In upper-class individuals, dispositions to embrace healthy and exotic food facilitate the adoption of new flavors and reflexivity in eating practices. Conversely, lower-class individuals have traditional meatless culinary practices rooted in their restricted budget, facilitating the transition to a plant-based diet.

**Discussion:**

These results demonstrate the relevance of social class in understanding the diversity of vegan practices, and they contribute to breaking stereotypes around this movement.

## 1 Introduction

Veganism is an ethical position, a political movement, and a lifestyle “which seeks to exclude—as far as is possible and practicable—all forms of exploitation of, and cruelty to, animals for food, clothing or any other purpose” (The Vegan Society, 2024). While individuals may embrace veganism for various reasons, including moral, environmental, and health concerns, they share a common practice: excluding animal products from consumption (Dutkiewicz and Dickstein, 2021). In fact, veganism is enacted through practices (Twine, 2017).

In recent decades, veganism has experienced a notable growth, with an increasing number of individuals adopting consumption practices, which exclude products of animal origin. The rise of veganism is exemplified by the participation in Veganuary, which increased from 400,000 participants in 2020 to 700,000 in 2023 (Veganuary, 2020, 2023). There has also been a considerable increase in interest in veganism, as reflected by a significant surge in Google Trends, which peaked during the COVID-19 pandemic (Google, 2024). In Chile, a survey suggests that 14% of the adult population identifies as vegetarian, while 4% identify as vegan (CADEM, 2018). This growth has gone hand-in-hand with a significant increase in the amount of research associated with the topic since 2019 (Salehi et al., 2023).

One of the characteristic elements of this movement is adopting a plant-based diet (Cherry, 2006; Gheihman, 2021), where individuals must relearn how to eat and cook while moving away from hegemonic culinary norms. Although people from various social classes choose this lifestyle (Giacoman et al., 2021), veganism is often perceived as a practice associated with the elite (Greenebaum, 2018). The objective of this article is to analyze how personal tastes of different social classes generate favorable (or unfavorable) dispositions to adopting veganism, placing special emphasis on culinary practices and taste as a critical point of convergence. This is especially relevant in Chile, a country that exhibits significant economic and cultural distinctions among its various social classes. Numerous studies indicate that Chilean society is deeply divided (López and Miller, 2008), particularly in its capital, Santiago. These variations are evident even in dietary habits and communal dining practices (Rivera and Giacoman, 2019).

In the field of food, the transition to veganism not only implies a new definition of what is and what is not edible (Giacoman et al., 2021) but it is also about learning new culinary practices (Twine, 2018). Previous studies show that adopting veganism involves culinary learning which includes new recipes, techniques, and ingredients, the veganization of known recipes, the use of substitutes and above all a re-education of taste toward a new vegan palate (Twine, 2018). However, continuity is also visible through techniques, recipes, and flavors of known preparations, without animal products, prior to the transition (Giacoman et al., 2023b).

It should be noted that the adoption of veganism does not occur in the same way in all individuals. In this article, we propose that social class of origin is crucial in understanding these differences. Veganism is commonly considered an elite practice (Greenebaum, 2018). This perception is based on the increased visibility of privileged vegans and a narrative that standardizes veganism around expensive, healthy, and exotic practices, such as the consumption of organic supermarket products, imported substitutes and the use of unusual kitchen utensils (Lee, 2017; Chatila, 2018; Greenebaum, 2018; Lindgren, 2020). In this way, it is assumed that the upper classes present a habitus with favorable dispositions toward the adoption of veganism (Ossipow, 1997), such as healthy, exotic, and cosmopolitan consumption (Kraaykamp, 2002; Warde and Gayo-Cal, 2009; Pulici, 2012; Bourdieu, 2016).

Countering this view, some authors have shown that veganism also develops outside of elites and that non-privileged vegans have specific ways of practicing veganism (Greenebaum, 2018; Giacoman et al., 2023a). Aligned with this critical position, this work shows that there are favorable dispositions in all social groups toward a transition to veganism, however, these dispositions will vary according to the habitus of the individuals' social class of origin. Thus, based on biographical interviews, this article contributes toward understanding the transition to veganism as a trajectory anchored in the diverse social origins, an element that is novel within the developed literature on the subject.

## 2 Literature review

Within the sociological study of food, social class has been a relevant element to understand the behavior of individuals. One of the most used theories to understand class differences is Bourdieu's (2016) theory of cultural capital and his concept of habitus. Bourdieu (2016) suggests that socialization (primary and/or secondary) makes up a set of dispositions expressed in practices and ways of thinking which allow individuals to be classified according to their position in the social field. Thus, different studies have shown that people from different social classes have different culinary tastes (Øygard, 2000; Kraaykamp, 2002; Tomlinson, 2003). Specifically, Bourdieu suggests that lower classes have simpler eating practices, associated with economic and temporal restrictions, categorizing it as taste of necessity (Bourdieu, 2016; Baumann et al., 2019). Therefore, lower classes are defined by consuming heartier and cheaper foods such as bread or French fries in Holland, Norway, and England (Øygard, 2000; Kraaykamp, 2002; Tomlinson, 2003) or legumes in Chile (Montecinos Aguirre, 2005; Aguilera Bornand, 2018). At the same time, they present a taste free of nutritional restrictions (Régnier and Masullo, 2009). On the other hand, the upper classes present a taste of freedom, presenting healthier and sophisticated eating practices, seeking distinctive consumption (Bourdieu, 2016). This is expressed by greater consumption of vegetables and by an appreciation for the variety in foods consumed (Øygard, 2000; Kraaykamp, 2002; Araneda et al., 2016; Oncini, 2019). Furthermore, cosmopolitan food consumption has become a form of distinction among the elites (Warde and Gayo-Cal, 2009; Rössel and Schroedter, 2015), accompanied by the preference for luxurious and exotic foods (Pulici, 2012). Studies from North America also shows that people with high cultural capital differentiate themselves through morality, such as ethical consumption (Carfagna et al., 2014; Kennedy et al., 2018). These studies show how class habitus have an impact on eating practices.

Taste can be visible in everyday practices. Practices constitute the foundation for shaping and reproducing everyday life, involving the interplay between individual agency and social structures (Shove et al., 2012). Reckwitz (2002) defines practices as routinized types of behavior comprising various interconnected elements: bodily and mental activities, the use of objects, underlying knowledge in the form of understanding, know-how, emotional states, and motivational knowledge. Therefore, we propose that vegan eating practices are an interaction of the primary class habitus with current vegan dispositions (Giacoman et al., 2023a).

Despite the importance of the habitus concept to understand dietary practices, there are few studies which explicitly incorporate the concept of habitus to analyze the adoption of vegan dietary practices. The first author to use the concept in the discussion is Ossipow (1997), in a study about the adoption of macrobiotic, vegetarian, and vegan diet. She argues that adherence to these diets implies the adoption of a secondary habitus, being more common among those who came from middle or high socioeconomic groups (Ossipow, 1997).

The project, which is the present work's frame, also uses the concept of habitus. Aligned with Ossipow's proposal that the adoption of a plant-based diet implies the adoption of secondary habitus, and following the contributions that Darmon has made on trajectories of anorexic women (Darmon, 2008) and socialization (Darmon, 2016), the project in its preceding publications proposes that the transition to veganism implies acquiring a gradual new habitus, shifting the understanding of the world and the practices (Giacoman et al., 2021). The primary author and colleagues identify a path in this transition consisting of five steps: personal questioning, vegetarianism attempt, vegetarianism, veganism, and activism. Although there are similar trajectories in adopting veganism, the social class of origin introduces nuances in the conversion process to this lifestyle (Giacoman et al., 2021). In another publication focusing on an online community of a low-budget vegan, the authors and colleagues show that the formation of the vegan habitus there is a process of individual construction that brings this new habitus into dialogue with its class dispositions of origin (Giacoman et al., 2023a).

The acquisition of new knowledge and culinary practices that make up this new vegan habit is carried out through secondary socialization (Ossipow, 1997). In other words, it refers to an apprenticeship at a juvenile or adult stage and implies a reconstruction of the previously formed individual identity to enter and adapt to new groups of social belonging (Darmon, 2016). This learning consists mainly of a search for nutritional information, watching videos, blogs and/or recipe books and sharing their experiences with other vegans (Elorinne et al., 2016; Lawo et al., 2020; Stano, 2021). Thus, various studies, without directly mentioning the concept of habitus, highlight the role of vegan peers in youth and adults, especially in social networks, when adopting vegan practices and beliefs (Ossipow, 1997; Cherry, 2015; Twine, 2018; Giacoman et al., 2021).

Vegan habitus, as any, is expressed in a series of classifications and practices. Regarding classifications, on the one hand, it redefines the edible and non-edible, guided by the defense of animal rights as an ethical principle (Giacoman et al., 2023b). This classification is so important that is even considered a symbolic boundary (Lamont and Molnár, 2002) for vegans and defines their authenticity (Greenebaum, 2012). On the other hand, is a cuisine which is highly aware of nutritional and medical food elements (Giacoman et al., 2023b). About practices, Twine (2017) and Giacoman et al. (2023b) describe practices associated with this new cuisine. Twine's works, employing Shove et al. (2012) framework, identifies three dimensions of vegan practice: materiality, competencies, and meanings. These meanings are primarily composed of motivations for veganism, such as animal exploitation and environmental concerns, alongside nutritional considerations for sustaining this lifestyle (Twine, 2017; Giacoman et al., 2023b). Moreover, Twine (2017) underscores the significance of cooking, culinary skills, nutritional knowledge and creativity as key competencies of vegan practice (Giacoman et al., 2023b). Finally, even though he does not use the concept of habitus, identifies four modes of material constitution in vegan practices: the substitution of ingredients, the exploration of new foods, culinary creativity, and new taste (Twine, 2018). Specifically, it is observed that the first three practices involve constant and active experimentation of taste (Twine, 2018). Thus, substitution as a practice allows maintaining dishes and flavors like previously consumed. On the other hand, exploring within this new cuisine with new condiments, techniques, and products, guides them toward variety, drawing a new vegan taste (Twine, 2018). Although there are few studies on how social class dispositions are coupled with the practices and principles of this new vegan habitus, there exists a prevalent association, both in collective and academic perceptions, linking upper classes with veganism (Harper, 2010; Deckha, 2012; Aguilar, 2015; Greenebaum, 2017; Lee, 2017; Aiswarya, 2019; Lindgren, 2020). Studies show that there is a narrative which standardizes veganism around expensive, healthy (Christopher et al., 2018), and exotic practices, such as consumption of organic supermarket products, imported substitutes and the use of unusual kitchen tools, reinforcing the idea of veganism as a practice of upper classes (Lee, 2017; Chatila, 2018; Greenebaum, 2018; Lindgren, 2020). Ethical consumption is also a practice linked with people with high cultural capital (Kennedy et al., 2018) due to the power of morality as a form of distinction (Lamont and Molnár, 2002). However, this narrative is not consistent with reality.

Faced with the overrepresentation of white and upper-class vegans, various studies have delved into the difficulties that lower-class vegans must face when transitioning. Roeder (2021) explains that there are certain urban sectors in North America, usually of low socioeconomic status, where the existence of fast foods based on meat and dairy prevails, making it difficult to find affordable vegan options. This link between meat consumption and popular groups is not only expressed in material restrictions, but also in the tastes of the individuals. Along these lines, Aguilar (2015), who studies food decisions in intentional communities in the United States, shows that popular and non-Caucasian groups have tastes associated with meat consumption (Aguilar, 2015; Lee, 2017). Thus, it is argued that there is a cultural limitation in which the palate in primary socialization adapts to non-vegan foods, so abandoning certain dishes or flavors becomes more difficult compared to more affluent classes (Aguilar, 2015; Lee, 2017).

This renunciation of the acquired taste in childhood, particularly in the context of the United States, is intensified by a shortage of vegan recipes that include flavors, ingredients or dishes, known in the primary socialization of non-hegemonic subjects, so the transition implies a renunciation of flavors and foods which were previously consumed (Aguilar, 2015; Greenebaum, 2018). Along these lines, the importance of seasonings used within vegan recipes is highlighted as being able to recover certain dishes, capturing the flavor and emotions of the “comfort food” of each individual (Greenebaum, 2018).

Contrary the compelling evidence in the United States regarding the association between upper-class and vegan habitus dispositions, and the existing difficulties for lower-class vegans, Giacoman et al. (2023a) carried out a study on a Chilean online community, revealing different results. The study describes the existence of a poor vegan habitus in Chile, highlighting how people manage to intersect their habitus of origin with the vegan one. Here, traditional dishes and the taste of necessity engage in a dialogue with foreign dishes and cosmopolitan tastes associated with the secondary vegan habitus.

Like most Latin American cuisines, Chilean cuisine is plural and changing (Giacoman, 2015). However, it tends to be characterized by the fusion of practices and foods consumed by the native peoples with those brought by the Spanish colonization (Montecinos Aguirre, 2005; Pereira Salas, 2007), which over time has also incorporated preparations and products contributed by multiple migrations (Giacoman, 2015). This mixture is visible in its characteristic ingredients that include potatoes, pumpkin, corn, beans, onion, and garlic, among others, present in several of its emblematic dishes such as *cazuela* (stew) or *humita* (tamal; Montecinos Aguirre, 2005). Meat is present in various dishes, but some preparations use only vegetables, such as *porotos granados* (bean and corn soup) and the *humitas* mentioned above (Giacoman et al., 2023a). Finally, it is worth mentioning that in Chile, meat consumption is frequent in all social groups, although it is in the lowest income quintile where we find a higher consumption of legumes (Crovetto, 2002).

This research continues to delve into the latest work exploring how people from different social classes origins have different dispositions toward veganism. We aim to understand the transition to veganism as a trajectory anchored in the diverse social origins, an element that is novel within the developed literature on the subject.

## 3 Materials and methods

The objective of this study is analyzing the dispositions of different social classes regarding the adoption of veganism. This research is contextualized in a larger research project (ANID FONDECYT N° 1201629) which has been approved by the social sciences ethics committee of Pontifical Catholic University of Chile (N° 190610025), both its objectives as well as all data collection instruments used for this study.

The process of data collection was performed over a period of 3 years, obtaining a total of 73 interviews from 40 different participants. The 1st year, in June 2020, an open call was made on social networks to recruit participants interested in sharing their experiences about veganism. During that year, a total of 30 people were interviewed. The 2nd year, in September 2021, contact was established with those participants who had been interviewed the previous year, obtaining responses from 15 of them. In addition, a second call was made through social networks to recruit 10 new participants. That year, the participants were asked to record a daily video 2 days a week, one on Wednesday and one on Saturday, in addition to the interview. Finally, the last year of data collection, in August 2022, the participants interviewed the previous year were contacted again, managing to obtain 18 interviews. In total, we conducted interviews with 40 vegans: 12 grown in upper-class families (30%), 14 grown in middle-class families (35%), and 14 grown in lower-class families (35%). The participant's details are in Table 1.

**Table 1:** Participants in the study.^*^

**Pseudonym**	**Gender**	**Age**	**Time in veganism**	**Social class of origin**	**Occupation of the parent with a higher rank**	**Occupation of the participant**	**Waves**
Josefa	Female	21	< year	High	Manager of a car company	Medical technology student	I
Pilar	Female	26	< year	High	Owner of a consulting firm	Lawyer	I
Valentina	Female	25	< year	High	Manager in human resources	Psychologist	I
María	Female	23	1 year	High	Doctor	Law student	I
Marisol	Female	26	3 years	High	Independent electric engineer	Law student	I, II, y III
Denisse	Female	25	4 years	High	Manager of a food company	Sociology student	I, II, y III
Consuelo	Female	27	7 years	High	Manager in a TV Company	CEO in an animal organization	I, II, y III
Elías	Male	20	2 years	High	University professor	Antropology student	I, II, y III
Elisa	Female	21	< year	High	International Trade Executive	Agronomy	I
Juan	Hombre	24	2 years	High	Architect	Anthropologist	I, II, y III
Rocío	Female	26	< year	High	Principal in a private school	Audiovisual specialist	I y II
Martina	Female	27	< year	High	Civil Engineer	Special education teacher	II
Carla	Female	22	< year	Middle	Truck owner	Business administration student	I
Guillermo	Male	28	< year	Middle	Salesmen for transportation	Architect	I
Sandra	Female	22	7 years	Middle	Taxi driver	Dietetics and nutrition student	I
Alejandra	Female	24	< year	Middle	Journalist	Nurse student	I y II
Viviana	Female	21	< year	Middle	Bank account executive	English translation student	I y II
Magdalena	Female	28	< year	Middle	Truck owner	Marketing professional	I, II, y III
Ana	Female	22	< year	Middle	Library manager	English teacher	I y II
Gloria	Female	27	8 years	Middle	Auditor accountant in a bank	Lawyer	I, II, y III
Claudio	Male	27	2 years	Middle	Truck owner	Lawyer	I, II, y III
Catalina	Female	26	3 years	Middle	Furniture craftsman	Medical technologist	I, II, y III
Rosa	Female	27	< year	Middle	Nurse technician	Odontology student	II y III
Margarita	Female	25	5 years	Middle	Library manager	Social worker	II
Raúl	Male	29	3 years	Middle	Saleswoman in a cosmetics store	Architect	II y III
Jennifer	Non Binary	21	3 years	Middle	Taxi driver	Veterinary student	II y III
Sebastián	Male	31	17 years	Low	Construction worker	Engineer in administration	I
Luisa	Female	27	2 years	Low	Security guard	International commerce	I
Paz	Female	23	8 years	Low	Construction worker	Occupational safety	I
Luis	Male	28	6 years	Low	Domestic worker	Nutrition student	I
Sofía	Female	28	2 years	Low	Welder	Insurance company	I
Katherine	Female	27	< year	Low	Construction worker	Elementary school teacher	I
Isadora	Female	35	< year	Low	Construction worker	Nutritionist	I
Simone	Female	35	2 years	Low	Domestic worker	Auditor accountant and artist	I, II, y III
Nicolás	Male	27	8 years	Low	Junior in a mining company	Lawyer	I, II, y III
Isidora	Female	26	2 years	Low	Machinery operator	Lawyer	II y III
Patricia	Female	27	3 years	Low	Craftswoman	Chef and Food Scientist	II y III
Trinidad	Female	21	3 years	Low	Salesperson in retail	Sociology student	II y III
Soledad	Female	23	< year	Low	Construction worker	Master's student	II y III
Pablo	Male	19	< year	Low	Salesmen in a small store	Kinesiology student	II

Source: Based on table's participant in Giacoman et al. (2023b) with additional information.

^*^All study participants resided in urban areas and the Metropolitan Region of Santiago, Chile, when they were selected to participate.

The sampling strategy was based on two selection criteria. First, the length of participants' involvement in veganism was considered, distinguishing between those with < 1 year of experience (initial) and those with more than 1 year of experience (consolidated). Secondly, the social class of the parents was considered according to Goldthorpe's occupational categories, with 10 participants per social class in each wave. According to Torche and Wormald (2004), in Chile, occupation delineates current and future opportunities for the majority. Given its strong correlation with two key dimensions in determining social stratification, educational level, and income, it serves as a reliable indicator of social class (Torche and Wormald, 2004). Therefore, occupation is strongly related with cultural capital. Using occupational categories, we can assume the educational level of each individual's family (i.e., professional categories usually have university degrees, whereas routine occupations usually have up to high school degrees).

It is worth noting that all participants are university students or have a university degree; hence, most individuals raised in lower- or middle-class families likely have more cultural capital than their parents. Therefore, the results must be considered carefully because adopting new vegan habits can be linked with the cultural capital acquired in university. Giacoman et al. (2021) state that social networks created at university are relevant in adopting and maintaining veganism.

Data collection was carried out through in-depth interviews as the main collection instrument. The first series of interviews focused on the life history of the participants and their transition process toward veganism. The second series included a detailed description of participants' current lives, including aspects related to their food, cooking, health, consumption, and activism around veganism. The video diaries, on their part, showed what had been consumed that day (on Wednesday and on Saturday) describing the dish, its preparation, its purchase as well as where and with whom it was consumed, and a reflection about the most important food. On the final series of interviews, the participants' current daily lives were explored and contrasted with information gathered in the previous interviews. The opinions and actions of the participants as regards veganism and consumption were investigated.

It is important to highlight that both, the interviews, and the data analysis, were carried out by the researchers themselves, which allowed a deeper immersion into the phenomenon and data comprehension. Furthermore, thanks to the flexibility of the interview schedule, the researchers were able to delve deeper into relevant topics of the research.

Due to the restrictions imposed by the COVID-19 pandemic, the interviews of the first two series were performed online, using the Zoom platform. As for the last series of interviews, the option arose to conduct them both online as well as in person, depending on the preference of the interviewee. During the online interviews, an external recording device to the platform was used to avoid recording the video.

In ethical terms, all participants signed an informed consent prior to conducting the interviews. We use pseudonyms to ensure participant's anonymity. Additionally, the researchers and transcribers signed a confidentiality agreement, ensuring that the information obtained would not be disclosed outside the purposes of the research. The interviews were stored in a shared folder with exclusive access to the project researchers, and it was established that said recordings and transcriptions would be deleted after 10 years of the completion of the research. The interviews were transcribed in Spanish and analyzed in Spanish.

We performed a thematic analysis inductively using the MaxQDA program, where codes were assigned to the relevant quotes, which were grouped successively until the identification of major themes is reached. This process was performed for each wave. Then, the codes corresponding to culinary learning and current cooking practices were identified, comparing the codes obtained for each series. Subsequently, the obtained codes were discussed among the research team and their possible interpretation.

## 4 Results

The following section presents the results in two sections. The first describes acquiring a new vegan taste, and the second explains how vegan taste intersects with the primary habitus. These sections are presented separately for analytical purposes, yet it is crucial to acknowledge that this process is simultaneous.

### 4.1 Retrain the taste

Veganism is expressed in a new classification of edible and inedible foods, excluding from the diet foods commonly consumed in omnivorous cuisine, such as meat, milk and cheese (Cherry, 2006). These new classifications lead to new daily eating and consumption practices, redefining a new lifestyle (Twine, 2017, 2018; Giacoman et al., 2023b). This involves learning a new cuisine and a taste consistent with this new adopted lifestyle.

Field work shows us that vegan taste has two great characteristics. On the one hand, it is a taste that is re-educated through new practices, learning new flavors (Twine, 2018) and even rejecting old flavors. On the other hand, we also find that in the vegan palate there is continuity, where they seek to imitate flavors valued in the past, which often maintain coherence with the new taste (Twine, 2018).

As previous studies have mentioned, the substitution of ingredients, the exploration of new foods and culinary innovation make the adoption of a new vegan taste necessary (Twine, 2018). Those interviewed highlight that due to the elimination of products of animal origin, “veganism forces you to create very different things in the kitchen,” and therefore, constantly experiment with new preparations and flavors. This is what Trinidad tells us. Trinidad is a sociology student who grew up in a lower class home; her parents work as a retail worker and a bus organizer in a bus depot. She highlights the inclusion of tofu in her diet and the process of learning to season it:

“It goes hand in hand with understanding the flavors of vegan food. This desire to imitate meat…, there are people who end up using too many seasonings and sometimes I feel that there is no need…, or sometimes you have to get familiar with the taste. It happened to me with tofu, which initially I found very bad, then it was like…, ‘same tofu with a little seasoning is good' and now I could even eat it alone, I find it delicious, so I feel that it takes getting used to experiencing new flavors (…), just the same, Chilean food is super limited in that sense (…) I have been trying to unlearn those things.”

Trinidad exemplifies for us how new practices, specifically the incorporation of tofu into her diet, learning to cook it and the associated culinary exploration, have shaped her new taste. This new taste consisted of a process of adaptation to the new flavor of tofu until it became familiar and even “good.” Thus, Trinidad highlights that part of introducing a new ingredient and preparation to the diet consists of understanding the new ingredient, both its flavor and consistency, and thus being able to season it according to its nature. In order to appreciate these new flavors, the interviewee tells us that the tastes acquired in primary socialization must be unlearned, acquiring a re-educated palate. Catalina, a medical technologist, doughter of a domestic worker and a furniture maker, explains to us that “one has had to re-educate one's palate, you have no choice, there are flavors that you will never be able to imitate 100%.” The interviewees highlight that the re-education of taste is particularly necessary in the country since Chilean cuisine often considers meat as a constituent element of the dish.

Reeducation of the palate is even presented as an aversion to products of animal origin such as milk or meat, excluding them from their consumption imitations that achieve a very similar resemblance to these products, both in flavor, appearance and texture. This is highlighted by Jennifer a veterinary student who grew up in a middle class home, mentioning that she does not like “things that are similar to meat, those that drip beet juice and seem to be blood, I don't like it (…) I like the flavor of vegetables, the flavor of legumes.”

Vegan taste brings with it a disposition toward culinary experimentation and innovation. Thus, vegans tell us how they have been incorporating ingredients, preparations and seasonings into their diet, especially from international cuisines (Giacoman et al., 2023b). Magdalena in her video diary shows a lunch preparation of whole grain noodles, with a vegetable sauce, coconut cream and crispy chickpeas. Crispy chickpeas are baked chickpeas, which were seasoned with turmeric, paprika and garlic powder. She added nutritional yeast to the final dish to deliver cheesy flavor. When reflecting on her diet for the day, she tells us that:

“The most important meal was lunch, it was whole grain noodles with vegetables and crunchy chickpeas (…) that dish reflects how diet has changed at home, with my family. When my mother cooked or even until now, it doesn't even cross her mind to eat whole wheat noodles with chickpeas. That's why I think it's important, it reflects in a little way, the type of food, dressings and products that I have been learning to cook.”

Thus, Magdalena shows us how culinary innovation is far from her previous cuisine and has been shaping her current cuisine. Magdalena is a buisness adinistrator that learned how to cook asking her mom, a stay-at-home mom, and later learned about vegan cuisine through friends, colegues and social media. This quote allows us to summarize what this culinary change implies for the interviewees and how these new tastes come into tension with those learned in primary socialization. Thus, this dish shows the incorporation of new ingredients, such as coconut cream and nutritional yeast. It also shows the incorporation of new seasonings, such as turmeric, and the creation of new preparations, such as the use of chickpeas as side dish to a plate of noodles. In addition, it shows us the incorporation of new proteins, such as chickpeas in a noodle dish, which is normally accompanied by meat or, failing that, soy meat, but rarely chickpeas. Thus, for Magdalena this dish shows how her current cuisine has been changing, crystallized in her new vegan habitus, one that uses ingredients unthinkable for her mother, both in their use and in their combination.

Magdalena's quote also shows us how this vegan taste also presents continuity with the previous taste. Thus, for example, nutritional yeast, although it is presented as a new ingredient, its functionality is to maintain the flavor consumed in previous preparations of noodles, imitating the flavor of grated cheese. There are tastes from the past which are consistent with the new taste. In continuity is where it is possible to see the weight of social class of origin, and the existence of class dispositions which align with vegan taste in food. Point to be developed in the following section.

### 4.2 Veganism and social class dispositions

Field work shows us that although everyone has to make a transition to this new taste, there are different facilities depending on social class of origin. This is because class-of-origin dispositions, both in practices and tastes, fit differentially with the vegan lifestyle. Thus, primary socialization is key to understanding current forms of vegan cooking.

#### 4.2.1 Restricted resources and taste of necessity

From the interviews we can observe that need is a transversal element when listening to the stories of popular groups. This is expressed both in practices, contextualized in scenarios of economic and temporal constriction, and in tastes associated with traditional recipes (Bourdieu, 2016; Baumann et al., 2019). Firstly, early learning as a result of necessity is evident, mostly presenting a first approach with salty dishes. Although the majority of the vegans raised in a lower-class home expressed an interest in helping and learning in the kitchen, the reason for learning to cook was commonly due to the lack of the adult figure who normally cooked at home. This is what Patricia, a lower-class vegan, who learned to cook when her grandmother died, tells us:

“*We learned to cook more out of necessity, because my grandmother died and it was like ‘we have to eat'… and not that my mother was the great cook…, my mother and I learned to cook more out of necessity than anything else, so these first steps were like… ‘mix this,' ‘grind it, now pour it in,' a functional thing.”*

Patricia tells how her first approach to cooking was not in a recreational space, but rather helping her mother prepare meals, something “*functional.”* Along these lines, the foods and preparations consumed at home remained in an established repertoire, limited to the culinary knowledge of the person in charge of cooking, commonly mothers. This is consistent with what the literature calls the sexual division of labor of food and household chores (DeVault, 1994; Cairns and Johnston, 2015). Even though Patricia is a chef and a food scientist nowadays, she still likes functional food. When we asked for her star dish, she chose rice, a recipe that learned the basics from her sister but perfected with time, innovating through trial and error. Soledad tells us that “*everything I learned was by asking my mother, because back then we didn't have internet, so it was simply by word of mouth.”* Thus, culinary knowledge was transferred orally and in writing. From a young age, lower class people are exposed to popular cuisine preparations, reproducing nowadays the flavors and culinary knowledge of their mothers.

Learning to cook at an early age becomes a facilitator when transitioning to veganism. This is because, as previous studies mention, cooking skills are a fundamental element in the constitution of vegan practice (Twine, 2017). Furthermore, due to the high prices and the time required to obtain certain products economically, one of the strategies most used by vegans of lower-class houses is to cook these foods at home. Along these lines, many popular vegans prepare their own hamburgers, milk, seitan, and among others.

The element of necessity when learning to cook is also expressed in this cuisine to which they are exposed. Those interviewed comment that although their families have always eaten meat, it is not consumed very frequently since it has a high economic cost. This is what Katherine tell us, pointing out that meat was considered a luxury in her lower-class home:

“*(In my family) they have always eaten meat, but it's not that much either because I don't come from a well-off family, so it's like meat is expensive, it's always been expensive, … My mom is very homely, cooking…, ever since I was little, beans, lentils, but every now and then she also indulges in eating some meat*.”

As Katherine tells us, excluding meat from the diet is cheaper. This is also mentioned by Sebastián, a son of a construction worker and a stay-at-home mom, who points out that a kilo of legumes is much cheaper than a kilo of meat, “and with that kilo of legumes I was able to make many different preparations, and I was able to eat perfectly for several days. Confirming the literature, due to its low economic cost to and high nutritional performance, we observe that legume stews (Montecinos Aguirre, 2005) and soy meat are foods that are consumed daily by the popular classes since childhood.

The taste associated with popular cuisine and the knowledge of its preparations facilitate the transition to the secondary vegan habitus when eliminating products of animal origin. Sebastián, raised in a lower-class home, is nowadays an engineer in administration, but still applies economic strategies to reduce the cost of food and to show other vegans through Instagram that eating vegan is not expensive. This also happens to Nicolás, who tells us that currently he is not “*a person with eccentric tastes, my plate of food is simple, beans, lentils, and with that I have enough, I am not interested in anything else.”* Nicolás' father lived in the countryside and used to eat legumes due to economic constrains, dishes that are reproduce in Nicolás' taste.

The cuisine of vegans raised in popular-class homes mixes necessity-oriented cuisine and the culinary innovation, characteristic of vegan cuisine (Bourdieu, 2016; Twine, 2018; Giacoman et al., 2023a), expressing itself in their actual taste. Isidora, a law student, daughter of a machinery operator and a secretary of a rug store, tells us that her current favorite dish is legumes, a preparation she learned at the time of her independence, to which she has incorporated variations according to her current taste:

“*At home we always cooked [legumes], so when I was there, I learned to cook them. When I left home, I had doubts about a particular food and I asked my mother: ‘When you prepare chickpeas, what do you add first?' That's how I saw how she prepared her dishes (…). At home they used oregano and salt, but later a friend said ‘oh, I love curry' and she added curry to everything and I got a taste for curry for some preparations, and another friend added pepper, so, just by cooking with other people you try the different tastes and it works for you. Now, I buy all sort of seasonings because I like to add a couple of them to my preparations, so that even though it Is the same dish (legume stew), it has a different flavor.”*

Isidora places great importance on the new seasonings discovered thanks to her friends, such as curry or pepper, which were not used by her mother and which she has begun to incorporate into her cooking. The interviewee mentions that she uses different seasonings, highlighting their power to make each dish different, even if the main ingredients are the same. Thus, although it seeks to replicate the dish, it does not do so exactly, the seasonings provide the element of variety and innovation sought in vegan cuisine.

The combination of both habitus in the current cuisine of popular social classes, is materialized above all by the veganization of dishes consumed in childhood. A good example of this is the preparation of Chorrillana, as shown in a diary video recorded by Simone, an accountant and artist raised in a lower-class home. Chorrillana is a typical dish from Chile and Peru, which consists of French fries with different types of meat, sausages, etc. along with onion and fried eggs. To replace meat and eggs, Simone uses shiitake mushroom, Chinese seaweed, pea meat, onion, tofu, and spices such as turmeric, curry, pepper, and black salt (for the egg flavor). Most of these elements, except for the onion, are new ingredients not used within this dish, and perceived as foreign to the traditional Chilean cuisine, such as shiitake mushroom, Chinese seaweed, and tofu.

Thus, this dish shows a fusion of Chilean culinary tradition and the new vegan palate. On the one hand, the structure of the Chorrillana with French fries, but topped with new varied ingredients corresponding to vegan cuisine. Simone also uses cornstarch to prepare a sauce. This is widely used, especially in lower classes, to thicken sauces more economically. Along these lines, although an adaptation of Chorrillana was prepared using ingredients associated with veganism, it also uses strategies learned in primary socialization, in order to grant texture.

#### 4.2.2 Freedom to explore and body ideals

Regarding the taste of vegans from wealthy households, we observe that they have practices associated with exotic consumption, expressed above all by the consumption of international cuisines, and a taste for what appears healthy and light (Øygard, 2000; Kraaykamp, 2002; Pulici, 2012; Bourdieu, 2016). It becomes evident that they did not experience the need as a decisive element in their cooking learning process. On the contrary, we observe vegans raised in upper-class homes have their first approach to cooking in moments of leisure, recognizing a taste for freedom (Bourdieu, 2016). Thus, the greatest motivation is self-interest, as Elías, an anthropology student son of a university professor and a consultant, tells us:

“*I remember that when I was younger, we had a maid who used to come every day and she would cook delicious dishes for us. There was not a bit of need to learn to cook either, my need arose when I would come home very hungry, after a night out and wanted to eat something tasty with good flavor. So around that time I asked the old lady ‘hey, I would like to cook,' and … I actually asked ‘hey, how did you cook this chicken?' so she showed me how to add orange to the chicken, which I think is very clever”*

Elías tells us how his need arose when he wanted to eat “something that tasted good” outside of the established food consumption hours, since these were covered. Thus, he begins to become interested in cooking, learning from observation and questions to the maid in charge of the kitchen, without getting directly involved in helping in the kitchen. Elía” quote shows us an early exposure to flavors far from the traditional cuisine, such as orange chicken, a dish from a foreign cuisine, awakening his interest in culinary innovation and international food, evidencing his cosmopolitan consumption (Warde and Gayo-Cal, 2009). Elías also tells us that this interest in cooking was also enhanced by the father of a friend who is a chef by profession and by watching YouTube videos of international chefs, developing a great technical knowledge around the subject. Along these lines, the taste for freedom of the upper classes is related to exposure from a young age to different flavors and types of preparations, and thereby, being more predisposed to trying new flavors.

Thus, when asked about their favorite dishes to prepare, they mention dishes such as Ramen, seitan, “stir-fried vegetables with oriental-type tofu” or gnocchi, all close to international cuisine. Early exposure to these flavors and preparations is a facilitator when transitioning to vegan cooking. This is what Pilar, a lawyer who grew up in an upper-class home, tells us, who highlights that international cuisine is very present in her family kitchen:

“*We prepare everything, we cook stews, we make ratatouille, we cook, I do”t know, like Indian food, we prepare dishes from all over the world, we try to incorporate legumes and vegetables in very different ways. So, actually being vegan or vegetarian was as easy as simply changing the cheese that was put on top and tha”s it.”*

Pilar's parents have university degrees, her dad owns a consultant company and her mom studied to be an accountant but is a stay-at-home mom who is in charge of cooking in the house. In Pila”s quote we can see that the freedom to explore was always part of her kitchen, where ingredients such as legumes or vegetables were cooked in a diverse variety of dishes and flavors. On the other hand, we observe that vegans from wealthy households use products that are more difficult to access in their preparations or veganization of dishes. This is what Juan, an anthropology student raised in a home with high economic and cultural capital, who uses agar-agar along with other binders to make ice cream, comments:

“*I buy, I do”t know, agar-agar, nutritional yeast, cmc which is like a polymer which is a binder, so as guar gum and, xanthan gum which is also a binder, dyes which I sometimes use when I make pastries, eggI. like not-egI(…) I usually make ice cream at home and when you use the binders t'ey don't have any flavor, but sometimes our intestines can reject them anyway, so the suggestion is ‘Pour a little bit of each.”'*

The agar-agar mentioned by Juan is a product used in vegan cuisine as a gelling agent, replacing gelatin, coming from animal cartilage. However, this is not widely used in Chile and is described by several Chilean vegans as a difficult-to-access and high-class product, while the other binders mentioned are not even among the cooking options (Giacoman et al., 2023a).

The use of uncommon and difficult-to-access ingredients makes the transition easier for them since they do not have economic restrictions when learning new preparations. This is mentioned by Consuelo, a CEO in an animal organization, daughter of a Manager in a TV Company and an alternative therapist, who recognizes that her “*privileged”* position has helped her avoid possible economic obstacles associated with the transition:

“*In my case I am super privileged, lik' I don't have financial problems, and at least in this process' haven't had them, so I have never felt like it is more expensive. But I do know that I consume some products which are considered as luxury, you get it? like cheeses from abroad, emmm, or certain sweets which are also more exclusive, eeehh, and I know that for someone else they could be an obstacle, but the truth is that't hasn't been for me.”*

Specifically, Consuelo defines imported foods as “exclusive” and “luxury” products, highlighting their high price. Along these lines, the consumption of imported products generates a distinction, associated with upper class habitus (Pulici, 2012; Bourdieu, 2016). Thus, she recognizes this possible difficulty for other vegans, emphasizing that thanks to her socioeconomic situation she can access these items without difficulty.

We also evidence that upper classes have a taste for light and healthy food (Øygard, 2000; Kraaykamp, 2002; Bourdieu, 2016), making it easier when transitioning to veganism. Vegans raised in upper-class homes claim to have had healthy food in their childhood, defined by very little fried foods and “lots of salad.” Along these lines, the consumption of fruits and vegetables to which they are accustomed also generates certain facilities. The concern for balanced consumption is accentuated in those who practice sports, focused on being able to adequately replace proteins. Marisol, a law student daughter of a nutritionist and an electric engineer, tells us that she has had an interest in healthy cooking even before she was vegan. This was a facilitator for her, especially when she started veganizing recipes of cakes and other sweets:

“*I started trying to dabble with healthy food issues, ‘healthy' (in quotes), trying to reduce sugar, or butter and things like that…, because by then'I wasn't vegetarian or vegan. I started preparing some healthy foods for myself and this was important for me. I also learned how to cook this type of healthy food, which made it easier later when I started replacing some products, because I was not starting from scratch, but rather understanding what it is that I am replacing when changing ingredients.”*

Marisol tells us how before becoming vegan she had an interest in healthy cooking, associated with reducing sugars and fats. This learning was key to her transition, since by needing to replace unhealthy ingredients, it made it easier for her to understand their functionality in preparing cakes and sweets. Thus, she uses the skills learned in her adolescence when transitioning to veganism.

#### 4.2.3 Restricted exploration

Regarding vegans raised in middle class homes, we observe that there are practices and tastes that reveal common elements with both the lower classes and the upper classes. On the one hand, monetary constraints and a taste of necessity are shown, especially associated with primary socialization, while on the other hand, an exploration associated with more occasional international cuisine and a concern for the light body is observed and a nutritionally valid diet.

About the cuisine learned in childhood, we observed that middle class houses had monetary restrictions similar to those of the lower classes, where meat was not a common meal because of its price. The relationship between economic consumption and exclusion of animal products is such that Guillermo, who grew up in a middle-class home, son of a Salesmen for transportation Handler in a food processor, tells us that his first motivation for being vegetarian was that it was less expensive. Thus, it is not a major complication to stop consuming foods of animal origin such as “cheese, ham, sausages and meat, because they were not a typical food at home.” Instead of meat, legumes and soy products were part of their daily diet. This is what Rosa, a daughter of a nurse technician, tells us, pointing out that soy products have always been part of her culinary culture, so the transition was not complex:

“*In my house we always consumed soy products. So, soy meat and soy milk, those things, were part of my usual diet, thus this transition, replacing ground meat with soy meat, or soy milk, it was easy for me because it was part of my food culture (…) I remember when I was very young, observing my grandmother cooking soy beans and making milk.”*

Rosa tells us that she had been exposed not only to the flavors of these products but also to observing their preparation, which has made it easier for her to currently prepare her vegetable milk.

Regarding culinary exploration, we also identified that people who grew up in middle-class homes, although they consider international cuisine in their diet, prepare it when they have guests. This is what Magdalena, who prepares chickpea curry for special occasions, tells us:

“*(I usually prepare chickpea curry) For guests, for events, at Christmas, when some friends suddenly drop by, I cook that for them, because maybe what I eat on a daily basis I may find it exquisite for me, but it is still 0 sophisticated…., But I feel that this is sophisticated and more innovative, and well… I suddenly prefer to add different ingredients, like the same base but instead of chickpeas I add tofu”*

Magdalena considers chickpea curry to be a sophisticated and novel dish, which is far from her routine meals. Thus, she uses this dish to bring closer the vegan cuisine to acquaintances and surprise her guests. This phenomenon is something that also occurs in the lower classes, where although thanks to vegan cuisine they have learned dishes and flavors from other cuisines. They leave these preparations for special occasions when they wish to demonstrate that vegan cuisine is good and tasty, despite the difficulty in obtaining certain ingredients. However, within everyday life they maintain a less “*sophisticated”* repertoire.

Finally, several interviewees from middle-class backgrounds told us that they were concerned about a light body, losing weight, and nutritional control of their diet before transitioning to veganism. Ana an English teacher, daughter of a library manager, mentions that at home “*We never ate a lot of fried foods, things like that, everything was…. always' I don't know, super balanced…. Furthermore, when I was little, I also had problems with my weight, so I was always seeing the endocrinologist, the nutritionist.”*

The early medicalized view of food, demonstrated in constant visits to the nutritionist and endocrinologists, provides them with medical knowledge about the necessary macronutrients, which facilitates the change in diet when transitioning. The relationship with nutrition in middle- and upper-class families is evident in its use to justify and maintain the vegan lifestyle within the family. Alejandra, a middle-class vegan for 8 months, tells us that when she transitioned to veganism her mother told'her: “*'yes,' but you're not going to be a vegetarian if you don't go first to the nutritionist', so I went to the nutritionist and after 3 months I became vegetarian.”*

## 5 Conclusion

The objective of this article was to analyze the dispositions of different social classes regarding the adoption of veganism. The results show that all social classes exhibit favorable dispositions in their habitus that facilitate the acquisition of the new vegan taste.

The results indicate that the people raised in upper-class homes present a habitus with favorable dispositions toward the adoption of veganism (Ossipow, 1997), such as healthy, exotic, and cosmopolitan consumption (Kraaykamp, 2002; Warde and Gayo-Cal, 2009; Pulici, 2012; Bourdieu, 2016). The upper classes have early exposure to international and exotic flavors, which is maintained when transitioning to veganism. At the same time, before becoming vegan they were already concerned about nutritionally balanced consumption, which facilitates the transition in terms of nutritional knowledge. Lastly, prior knowledge of healthy preparations makes the transition easier. Thus, what was expected from the literature is confirmed.

On the other hand, the results show that not only the upper classes have favorable dispositions but also the lower and middle classes. Vegans raised in lower-class homes have traditional meatless culinary practices rooted in their restricted budget, facilitating the transition to a plant-based diet. In Chile, although meat is valued, its consumption is not common among the lower classes due to its high price, so eliminating it from the diet was not so complex. Furthermore, foods that constitute the Chilean lower-class home, such as legumes or soy meat, are consumed to this day by lower-class vegans, having been exposed to these flavors and preparations since they were children in their homes. These results show that lower-class vegans not only face difficulties when transitioning to veganism but also present favorable dispositions for the transition. Thus, they should not completely renounce old flavors, but rather redefine traditional dishes with new preparations, ingredients, and seasonings, mixing both habitus.

Regarding the middle classes, it is observed that there are common practices and tastes with both the lower classes and the upper classes. On the one hand, monetary constraints and taste of necessity are shown (Bourdieu, 2016), especially associated with primary socialization, while on the other hand, an exploration associated with international cuisine is observed, more occasional than the upper classes, and a concern for the light body and a nutritionally valid diet.

This article presents three contributions to the existing literature. Firstly, it uses the concept of habitus, which, unlike previous studies focused on the current practices of vegans, incorporates taste as a distinctive element within the transition. Secondly, it applies biographical interviews, addressing the habitus of origin within the analysis. Thanks to working with the dispositions learned at the origin, it allows us to approach the phenomenon of re-education of taste, more than just the current formation of taste and its expression. Third, evidence on veganism is generated from the global south by addressing the geographical gap identified by Salehi et al. (2023). In Chile, vegan products and dishes such as legumes have been linked to disadvantaged sectors (Montecinos Aguirre, 2005), shedding light on the fact that the adoption of vegan practices can be facilitated by lower class habitus.

Among the limitations of this study is that all the interviewees are studying at university or graduated from it, so the current cultural capital is high, even for those from a lower-class origin. This may cause those elements, such as cosmopolitan and light consumption, characteristic of veganism, appear stronger. Therefore, we work with individual origin habitus rather their current one. Future research could delve into how the different subjects were socialized in this re-education of taste.

Finally, in line with the works of Greenebaum (2018) and Giacoman et al. (2023b) this article demonstrates that veganism is not only a practice of people with high-class backgrounds but that people with lower and middle classes habituscan also practice it. The results show that veganism is not always a total break with the culinary tradition of lower-class individuals, but it even presents continuities.

## Data availability statement

The dataset (coding tree) presented in this study are available on request from the corresponding author. The data are not publicly available due to Ethics Committee rules.

## Ethics statement

The studies involving humans were approved by Ethics Committee of Social Sciences of Pontifical Catholic University of Chile (protocol code 190610025, 03/04/2020). The studies were conducted in accordance with the local legislation and institutional requirements. The participants provided their written informed consent to participate in this study.

## Author contributions

CG: Conceptualization, Formal analysis, Funding acquisition, Investigation, Methodology, Project administration, Writing—original draft. CJ: Formal analysis, Methodology, Writing—original draft.
